# Cataract-Causing Mutation of Human Connexin 46 Impairs Gap Junction, but Increases Hemichannel Function and Cell Death

**DOI:** 10.1371/journal.pone.0074732

**Published:** 2013-09-03

**Authors:** Qian Ren, Manuel A. Riquelme, Ji Xu, Xiang Yan, Bruce J. Nicholson, Sumin Gu, Jean X. Jiang

**Affiliations:** 1 First Hospital of Lanzhou University, Lanzhou, China; 2 Department of Biochemistry, University of Texas Health Science Center, San Antonio, Texas, United States of America; Emory University School of Medicine, United States of America

## Abstract

Connexin channels play a critical role in maintaining metabolic homeostasis and transparency of the lens. Mutations in connexin genes are linked to congenital cataracts in humans. The G143R missense mutation on connexin (Cx) 46 was recently reported to be associated with congenital Coppock cataracts. Here, we showed that the G143R mutation decreased Cx46 gap junctional coupling in a dominant negative manner; however, it significantly increased gap junctional plaques. The G143R mutant also increased hemichannel activity, inversely correlated with the level of Cx46 protein on the cell surface. The interaction between cytoplasmic loop domain and C-terminus has been shown to be involved in gating of connexin channels. Interestingly, the G143R mutation enhanced the interaction between intracellular loop and Cx46. Furthermore, this mutation decreased cell viability and the resistance of the cells to oxidative stress, primarily due to the increased hemichannel function. Together, these results suggest that mutation of this highly conserved residue on the cytoplasmic loop domain of Cx46 enhances its interaction with the C-terminus, resulting in a reduction of gap junction channel function, but increased hemichannel function. This combination leads to the development of human congenital cataracts.

## Introduction

The crystalline lens is a transparent, avascular, biconvex structure in the anterior part of eye, which helps to refract and focus light on the retina. The lens consists of three parts, the outermost lens capsule, the interior lens fiber that forms the bulk of lens, and the lens epithelium, which is located between other two structures and only present on anterior side [1]. The fiber cells are linked to each other and connected with cells at the lens surface *via* gap junction channels, forming a large intercellular communication network. In lens, because of a lack of blood supply, gap junction coupling largely contributes to lens homeostasis and the maintenance of transparency [2]; [3]. Gap junctions are clusters of transmembrane channels that connect two adjacent cells and allow small molecules (M_r_≤1 kDa), such as ions, metabolites and second messengers, to pass from cell to cell [4]. Each gap junction channel is formed by two hemichannels, also called connexons, from two neighboring cells aligned with each other. Each connexon consists of six connexins, a family of membrane proteins containing 21 members in humans [5]. Connexins have four conserved transmembrane domains, two extracellular loop domains, one intracellular loop domain, and cytoplasmic NH_2_- and COOH-terminal domains. Compared with conserved transmembrane domains, extracellular loop and cytoplasmic NH_2_-terminal, the intracellular loop and COOH-terminal domains are highly variable between family members.

Cx46 and Cx50 are predominantly expressed in lens fiber cells [6]. Cx46 plays a critical role in coupling of fiber cells, especially in mature fiber in the central core of the lens. Deletion of the *GJA*3 gene (encoding Cx46) leads to severe nuclear cataracts in mice [7]. Increasing evidence over the last decade suggests that mutations in the Cx46 and Cx50 encoding genes, *GJA*3 and *GJA*8, respectively, are directly linked to human congenital cataracts in North and Central America, Europe and Asia [2]; [8]. Interestingly, most of the connexin mutations associated with cataracts are located in transmembrane and extracellular loop domains, including the 20 cataract-associated mutations of Cx46 [9]. One exception, the missense mutation G143R located in the cytoplasmic loop domain of Cx46, was present in a four-generation Chinese family with congenital Coppock cataract [10], a type of cataract described by Nettleship and Ogilvie in 1906 [11]. The opacity of this type of cataract is in the posterior region of the lens between the nucleus and the posterior pole. Gly-143 is a highly conserved amino acid residue across members of connexin family from various animal species. In this study, we show that the G143R mutation increased the interaction between the intercellular loop domain and Cx46. This mutation leads to abnormal alteration of gap junction and hemichannel function, leading to decreased cell viability, a possible underlying mechanism for the formation of cataract.

## Materials and Methods

### Materials

QIAamp® DNA Blood Mini Kit, QIAquick®PCR Purification Kit and QIAquick® Gel Extraction Kit were purchased from QIAGEN, Hilden, Germany. QuikChange™ Site-Directed Mutagenesis Kit was purchased from Stratagene, La Jolla, CA, USA. Paraformaldehyde was purchased from Electron Microscopy Science, Fort Washington, PA, USA. Dulbecco's Modified Eagle Medium (DMEM), 0.25% Trypsin-EDTA solution, penicillin/streptomycin and Alexa Fluor 350 were purchased from Invitrogen, Carlsbad, CA, USA. Fetal bovine serum (FBS) was obtained from Hyclone Laboratories, Logan, UT, USA. Neon® Transfection System device and Neon® Transfection System 100 µL Kit and GENETICIN® G418 were purchased from Invitrogen. Chemiluminescence kit (ECL) was purchased from Amersham Pharmacia, Piscataway, NJ, USA. EZ-link™-Sulfo-NHS-LC-Biotin and NeutrAvidin® UltraLink Resin were purchased from Pierce, Rockford, IL, USA. Anti-human Cx46 antibody was purchased from Santa Cruz Biotechnology, Santa Cruz, CA, USA. Glutathione-agarose beads and L-glutathione reduced were purchased from Sigma. Cell proliferation reagent WST-1 was purchased from Roche, Mannheim, Germany. Propidium iodide (PI) solution was obtained from BioLegend, San Diego, CA, USA. Annexin V Apoptosis Detection Kit APC was obtained from eBioscience, San Diego, CA, USA. All other chemicals were purchased from Sigma or Fisher Scientific unless it is specifically mentioned.

### Subcloning of Cx46 DNA

The coding sequence from the exon 2 of the *GJA*3 gene was amplified from genomic DNA by nested PCR using the following pairs of primers: external primers (Sense: 5′-CCCCCATCCAGCACCCCTTCA-3′. Antisense: 5′-CCCCGCCACCCCCAAACTCA-3′); internal primers (Sense: 5′-GCAGAGGATCCGATGGGCGATGGAGCTTTCTGG-3′. Antisense: 5′-GCAGATCTAGACTAGATGGCCAAGTCCTCCGGTCTG-3′). The PCR products were purified using QIAquick® PCR Purification Kit and subcloned into pcDNA3.1 vector. The Cx46G143R mutant was generated using the QuikChange™ Site-Directed Mutagenesis Kit with the following primers: Sense: 5′-CAGGGTGCGCATGGCCAGGGCGCTGCTGCGGACCT-3′. Antisense: 5′-AGGTCCGCAGCAGCGCCCTGGCCATGCGCACCCTG-3′. The sequences of Cx46 and Cx46G143R were confirmed by sequencing at the University of Texas Health Science Center at San Antonio DNA Sequencing Facility. The Cx46 and Cx46G143R coding regions were isolated following digestion with *BamHI* and *NsiI* enzymes, and then subcloned into pIRES2-EGFP vector, which contains two promoters driving expression of GFP and connexin separately.

### Cell culture

HeLa cells were cultured in DMEM supplemented with 10% fetal bovine serum, 100 units/ml of penicillin and 100 µg/ml of streptomycin, 5% CO_2_ and 37°C. Cells were transfected with Cx46-pIRES2-EGFP or Cx46G143R-pIRES2-EGFP vectors using the Neon® Transfection System 100 µl Kit according to the manufacturer's instructions. Transfected cells were selected with G418 at the concentration of 1 mg/ml for 2 weeks. The FACSAria cell sorter (BD Biosciences, Germany) was also used to isolate EGFP-positive cells, as a marker of Cx46 or Cx46G143R expression. To maintain stability of expression of Cx46, 0.5 mg/ml of G418 were applied during cell culturing.

### Western blot

Crude membrane proteins from cells were prepared as previously reported [12]. Briefly, cells were collected in lysis buffer (5 mM Tris, 5 mM EDTA, 5 mM EGTA plus protease inhibitors, 20 µl/ml phenylmethylsulfonyl fluoride, 20 µl/ml N-ethylmaleimide, 10 µl/ml NaVO_4_ and 10 µl/ml leupeptin), homogenized and centrifuged at 100,000× g at 4°C for 30 min and resuspended in lysis buffer. Crude membrane proteins were separated by 10% SDS-polyacrylamide gel electrophoresis, transferred to nitrocellulose membranes and blotted with anti-Cx46 (1∶500 dilution) recognizing the C-terminus of human Cx46, anti-β-actin (1∶5000 dilution) and anti-GAPDH (1∶10000 dilution) antibodies. Secondary antibodies, peroxide-conjugated anti-rabbit or anti-mouse IgG, were detected by a chemiluminlunance reagent kit. The membranes were exposed to Blue X-ray films (Phenix, NC, USA) and detected by fluorography. The intensities of specific bands were analyzed and quantified by densitometry.

### Immunostaining

Cells were cultured on poly-D-lysine (10 µg/ml) coated glass coverslip. After reaching 80% confluency, cells were fixed by 2% paraformaldehyde for 30 min and blocked with blocking solution (2% goat serum, 2% fish skin gelatin, 0.25% Triton X-100 and 1% bovine serum albumin in PBS) for another 30 min. Human Cx46 proteins were labeled with rabbit anti-human Cx46 antibody (1∶100 dilution), followed by rhodamine-conjugated anti-rabbit antibody (1∶400 dilution). The nuclei were labeled with DAPI (1∶25000 dilution). The cells were observed by Olympus BH-2 fluorescence microscopy and the images were analyzed by NIH Image J software.

### Dye Transfer Assay

Untransfected HeLa cells and HeLa cells transfected with Cx46 and Cx46G143R were plated on 35 mm dishes to reach over 90% confluency. Cells were bathed with recording medium (HCO_3_^–^-free αMEM medium buffered with 10 mM HEPES). Cells were microinjected using an Eppendorf micromanipulator InjectMan NI 2 and Femtojet (Eppendorf) at 37°C with Alexa Fluor 350 (Invitrogen) (20 mM in PBS) and allowed to transfer for 5 min. Dye transfer was observed under an inverted microscope equipped with Xenon arc lamp illumination and a Nikon eclipse (Nikon, Japan) (excitation wavelengths 330–380 nm; emission wavelengths above 420 nm). The index of dye coupling was scored by counting the number of cells with dye coupling against total injected cells. The fluorescent intensity of Alexa Fluor 350 in donor cells and recipient cells was also measured and the ratios of recipient cell to donor cells were used to show the function of transfer by gap junction.

### Dye Uptake Assay

Dye uptake was evaluated using time lapse measurements or snap shot images. Transfected HeLa or un-transfected control HeLa cells were bathed in recording solution (154 mM NaCl, 5.4 mM KCl, 1.8 mM CaCl_2_, 1 mM MgCl_2_, 10 mM glucose and 10 mM HEPES, pH 7.4). For Ca^2+^- free condition, cells were washed 3 times for 5 min each with recording solution without CaCl_2_ and MgCl_2_ supplemented with 5 µM EGTA (pH 7.4). After that, the cells were exposed to Etd^+^ at final concentration of 25 µM for time lapse recording or 50 µM Etd^+^ for snap shot recording. In the time lapse recording, fluorescence was recorded at regions of interest in different cells with Nikon eclipse filter (rhodamine B filter) (excitation wavelengths 540–580 nm; emission wavelengths 600–660 nm). Images were captured with a CoolSNAP HQ^2^ fast cooled monochromatic digital camera (16-bit) (Photometrics, Tucson, AZ, USA) every 2 min (exposure time = 100 ms, gain = 1) and image processing was performed off-line with ImageJ software (NIH). The collected data was illustrated as ΔF, fold difference of initial fluorescence and fluorescence at the time of interest *versus* the basal fluorescence.

For snap shot images, cells were exposed to 50 µM Etd^+^ for 5 min or 500 µM Alexa350 for 10 min, rinsed 3 times with PBS and fixed with 2% formaldehyde. At least 3 microphotographies of fluorescence fields were taken with a 10× dry objective in an inverted microscope (Carl Zeiss) with a rhodamine filter. The intensities of Etd^+^ in cells were measured and quantified by Image J software.

### Cell Surface Biotinylation

Cell surface biotinylation assay was performed on the basis of the previously published method [12] with some modifications. Cells were cultured on 60 mm dishes till reaching over 90% confluency. Untransfected HeLa cells were used as negative control. The cells were then rinsed 2 times with cold PBS (containing 1.0 mM CaCl_2_ and 0.5 mM MgCl_2_) and labeled twice with 0.5 mg/ml of EZ-link™-Sulfo-NHS-LC-Biotin for 15 min each followed by washing 3 times with 15 mM glycine to quench biotinylation reaction and a final wash with glycine for 5 min. Cells were lysed in 500 µl RIPA buffer (25 mM Tris-HCl, 100 mM NaCl, 10 mM EDTA, 1% Triton X-100, 0.1% SDS in PBS plus protease inhibitors: 2% phenylmethylsulfonyl fluoride, 2% N-ethylmaleimide, 1% NaVO_4_, 1% leupepetin,). Cell lysates were sonicated and centrifuged at 12,000 g at 4°C for 10 min. The supernatants with same amount of total proteins were combined with 500 µl of 50 mM Tris-HCl, mixed with NeutrAvidin® UltraLink Resin and incubated at 4°C overnight. The beads were washed 3 times with RIPA buffer (without SDS) and twice with cold PBS. The biotinylated proteins were eluted by boiling for 5 min in 1×sample buffer (50 mM Tris, 1% SDS, 2% β-mercaptoethanol, 15% glycerol, pH 6.8). Based on the amount of pre-loading Cx46, the biotinylated proteins were compared and analyzed by NIH Image J software.

### Triton X-100 extraction assay

Transfected cells were cultured on 60 mm dishes at over 90% confluency. Triton X-100 extraction assay was performed using a modification of the method described previously [12]. Cells were rinsed with cold PBS 3 times, collected in lysis buffer (5 mM Tris, 5 mM EDTA, 5 mM EGTA and 200 mM sucrose with protease inhibitors and phosphatase inhibitors) and centrifuged at 140,000 g at 4°C for 30 min. The pellet was resuspended with 1% Triton X-100 in lysis buffer and incubated for 30 min. A quarter of the lysate was kept on ice as whole lysate samples. After 30 min, three-quarter of lysate samples were centrifuged at 140,000× g at 4°C for another 30 min. The supernatant was collected and labeled as Triton X-100-soluble fractions. The pellet was resuspended in lysis buffer with 1% SDS as Triton X-100-insoluble fractions. Cell lysates, Triton X-100-soluble and -insoluble fractions were loaded on a 10% SDS acrylamide gel and analyzed by western blotting. The intensities of bands were analyzed using densitometry by NIH Image J software.

### Expression and purification of GST-Cx46/Cx46G143R loop fusion protein and GST pull down assay

Cx46 and Cx46G143R intracellular loop regions were amplified from the constructs of Cx46 and Cx46G143R in pIRES2-EGFP vector by PCR using the following pairs of primers: Cx46 loop primers (Sense: 5′-CACAGGATCCCTGCACATCGTGCGCATGGA-3′. Antisense: 5′-CTTCGAATTCGGTCCGCAGCAGCGCCCCGGCCATG-3′); Cx46G143R loop primers (Sense: 5′-CACAGGATCCCTGCACATCGTGCGCATGGA-3′. Antisense: 5′-CTTCGAATTCGGTCCGCAGCAGCGCCCTGGCCATG-3′). The PCR products were purified and subcloned into pGEX-2T vector. DH5α cells were transformed with GST-Cx46/Cx46G143R-pGEX-2T plasmids and cultured at 37°C. IPTG was used to induce the production of GST-Cx46 and GST-Cx46G143R fusion proteins, which were affinity-purified by using glutathione-agrose beads. GST-Cx46, GST-Cx46G143R fusion proteins or GST alone were incubated with membrane proteins from cells expressing Cx46 on a rotating platform at 4°C overnight. The eluted proteins from the pull down were run on 10% acrylamide SDS gel and immmunoblotted with anti-Cx46 and anti-GST (1∶5000) antibodies.

### Cell viability assay with WST-1 reagent

WST-1 assay was performed according to the manufacturer's instruction. Cells were grown in 96-well plates with 100 µl culture medium in each well. Ten microliters of WST-1 reagent was added to each well and the plate was incubated at 37°C for 1 hr, before shaking thoroughly for 1 min, and the absorbance of each well was measured by microplate (ELISA) readers (Infinite 200). The absorbance of the well containing only 100 µl of culture medium and 10 µl WST-1 reagents was measured as a background control. The measure wavelength was 450 nm and reference wavelength was 690 nm. Cell viability was presented as a ratio of the absorbance at 72 hr versus 12 hr after plating.

### H_2_O_2_ treatment and cell viability by flow cytometry

Cx46 or Cx46G143R-transfected HeLa cells were seeded in 6-well plates and subjected to 500 µM H_2_O_2_ treatment for 12 hr after they reached approximately 95% confluency (or use sparse cultured cells). Cells and culture medium were harvested, based on the manufacturer's protocol. Cells were briefly washed with PBS and binding buffer, and then resuspended in binding buffer. Ten microliters of PI and 5 µl annexin V were added to the cell suspension, and were incubated at room temperature for 15 min. Samples were then kept on ice and analyzed by LSR-II flow cytometry (BD Biosciences, Germany).

### Statistical analysis

All data were analyzed by GraphPad Prism 5 Software (GraphPad Software, La Jolla, CA). Two group comparisons were performed using paired design t-test. Multiple group comparisons were conducted by One-way ANOVA and Newmann-Keul's multiple comparison test. The data were presented as the mean ± s.e.m. of at least three measurements. Less than 0.05 of *P* value was designed as statistic significance difference. Asterisks in all figures indicate the degree of significant differences compared to controls, ^*^, *P*<0.05; ^**^, *P*<0.01; ^***^, *P*<0.001.

## Results

### Cx46G143R mutation decreased gap junction coupling, but increased formation of gap junction plaques

**Figure 1:**
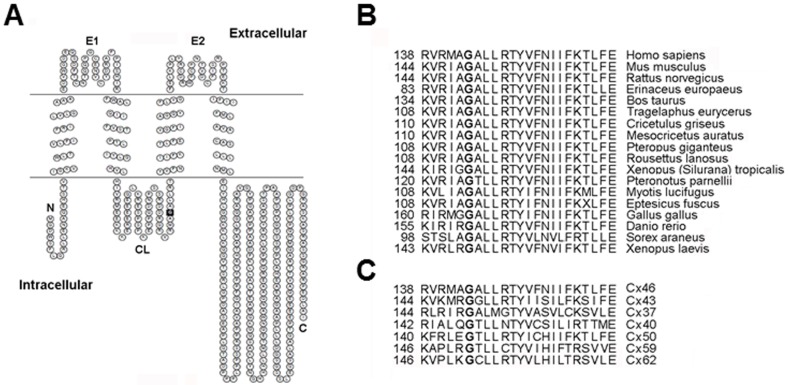
G143R mutation of human *GJA3* gene is located at the cytoplasmic loop domain of Cx46 protein. *A)* The membrane topological structure of Cx46 was generated by TOPO2. G143R mutation (indicated by solid black square) is located in the cytoplasmic loop domain. N, NH_2_-terminus; C, COOH-terminus; E1, first extracellular loop domain; E2, second extracellular loop domain; CL, cytoplasmic loop domain. *B)* Glycine at position 143 of Cx46 protein is highly conserved in Cx46 across animal species. *C)* Amino acid residue Gly-143 is also conserved in different human connexins.

The G143R missense mutation is located in the cytoplasmic loop domain of human Cx46 using three different topology prediction servers. The topological map of Cx46 was produced based on TMpred using TOPO2 (Fig. 1A). Glycine at position 143 is highly conserved among Cx46 orthologs across animal species (Fig. 1B) and also among different members of the human connexin family (Fig. 1C).

**Figure 2:**
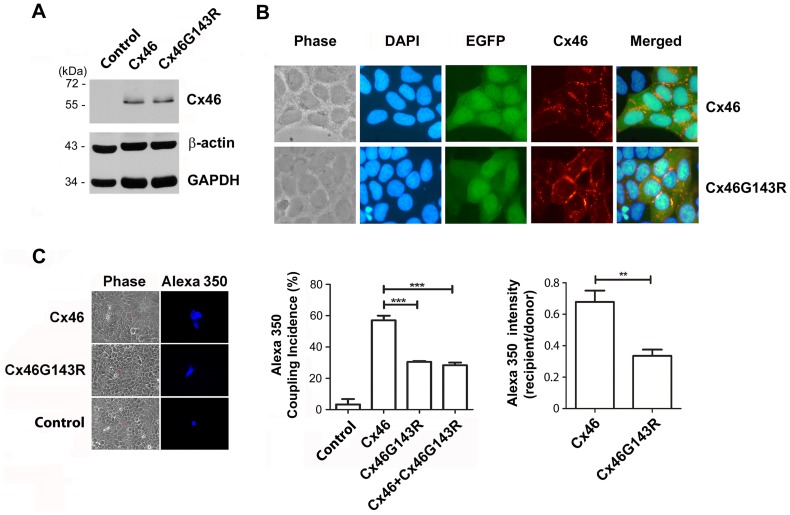
Cx46G143R, like wild-type Cx46, is localized at junctional plaques, but inhibits dye coupling in a dominant negative manner. *A)* HeLa cells were stably-transfected with wild-type Cx46 or Cx46G143R. Crude membrane extracts of parental HeLa cells (control) and stably transfected cells expressing wild-type Cx46 or Cx46G143R mutant were immunoblotted with anti-Cx46, β-actin or GAPDH antibody. *B)* Cx46 and Cx46G143R-transfected HeLa cells were fixed and labeled by anti-Cx46 antibody and followed by labeling with rhodamine-conjugated anti-rabbit secondary IgG and countered stained with DAPI. Wild-type Cx46 and Cx46G143R are localized at gap junction plaques. *C)* HeLa cells were untransfected (control) or transfected individually with Cx46, Cx46G143R or co-transfected with a 1∶1 of the plasmids. Forty-eight hr after transfection, cells were microinjected with Alexa Fluor 350 dye (20 mM). The cells were fixed 5 min after microinjection and the degree by dye transfer was quantified. Frequency of dye transfer was measured as the% of injected cells showing dye transfer to at least one neighbor. Untransfected HeLa cells were used as a negative control. Cx46G143R or Cx46G143R plus Cx46 versus Cx46, ^***^, *P*<0.001. n = 3 (∼15 cells/each experiment). The ratios of Alexa Fluor 350 fluorescent intensity in each recipient cell compared to the injected (donor) cell were calculated to show the efficiency of transfer by gap junctions, Cx46G143R versus wild-type Cx46,^**^, *P*<0.01.

To dissect the molecular mechanism behind this mutation that might lead to the disease phenotype, wild-type Cx46 or Cx46G143R mutant DNA constructs were stably transfected into HeLa cells. Similar levels of the expression of wild-type or mutant protein were detected by Western blot around 55 kDa, whereas no expression of Cx46 was detected in untransfected HeLa cells (Fig. 2A). Co-expression of EGFP allowed identification of transfected cells. Immunofluorescence analysis showed that both Cx46 and Cx46G143R proteins were primarily localized at the plasma membrane in large plaques at cell-cell interfaces (Fig. 2B). Low level(s) of wild-type Cx46 was also detected in the cytoplasm and this was more prominent for the mutant.

A microinjection dye transfer assay was performed to investigate the effect of CxG143R on gap junction coupling. Untransfected HeLa cells, or HeLa cells transfected with EGFP vector above were used as negative controls. Alexa Fluor 350 dye coupling was only observed in cells expressing Cx46 or Cx46G143R, but not in control cells (Fig. 2C). However, compared to wild-type Cx46 expressing cells, gap junctional coupling decreased by over 2 fold in Cx46G143R mutant-expressing cells, in terms of the frequency of dye transfer (Fig. 2C, middle panel) or its efficiency, assessed by the fluorescent intensity ratio of recipient to donor cell (Fig. 2C, right panel).

To determine if the reduced function of this mutant might have a dominant effect, HeLa cells were co-transfected with Cx46 and Cx46G143R at 1∶1 ratio. Intercellular coupling in cells co-expressing Cx46 and Cx46G143R exhibited similar levels of dye transfer to that in cells only expressing Cx46G143R, [i.e. about half of the level in wild-type Cx46 cells (Fig. 2C, middle panel)].

**Figure 3:**
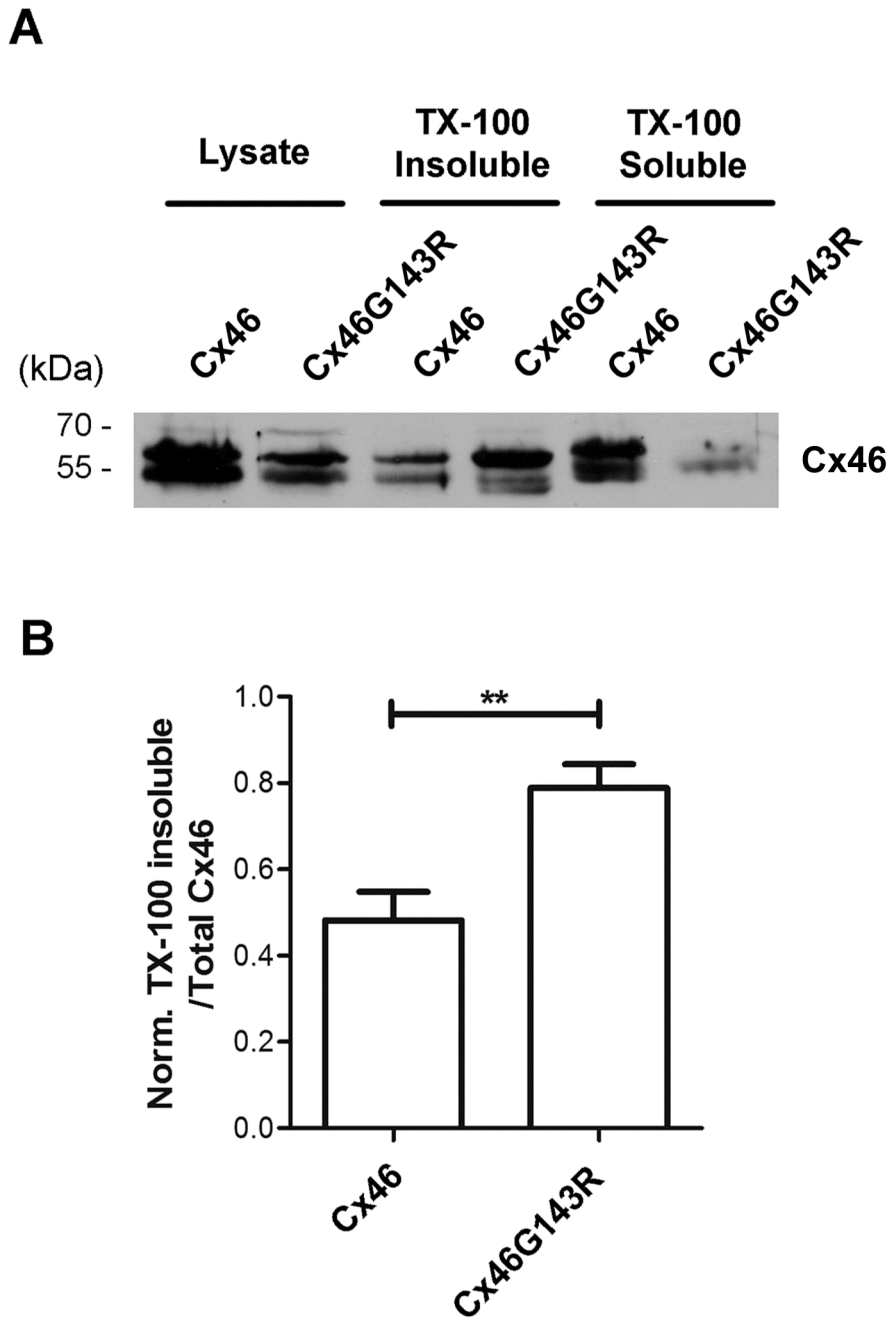
Cx46G143R enhances gap junctional plaque formation. *A)* Lysates of HeLa cells expressing exogenous wild-type Cx46 or Cx46G143R were prepared, and the Triton X-100-soluble and insoluble fractions were separated by centrifugation and analyzed by western blotting using anti-Cx46 antibody. *B)* The ratio of Triton X-100-insoluble fraction to total Cx46 protein was quantified using densitometry. Cx46G143R verses wild-type Cx46, ^**^, *P*<0.01. n = 4.

Cx43 gap junction channels are normally localized in detergent-resistant junctional plaques [13]. To test the possibility that the decreased gap junctional coupling in mutant expressing cells was caused by reduced formation of junctional plaques, we assessed levels of Triton X-100 detergent resistant forms of Cx46 from the different transfectants studied above. Consistent with observations from immunofluorescence studies (Fig. 2B), despite a lower level of Cx46G143R expression compared to wild-type Cx46, the mutant showed higher levels of protein in the detergent-resistant junctional plaque fraction than wild-type Cx46. This was best illustrated by the ratio of Triton X-100-insoluble to total Cx46 protein (Fig. 3). These results suggest that Cx46G143R enhances the formation of gap junction plaques. Furthermore, it implies that the reduced gap junctional coupling is likely to result from non-conductivity of the channels, and not the failure to assemble into stable gap junction structures, which appears to be enhanced.

### G143R mutation increased hemichannel activity, but decreased cell surface expression of Cx46

**Figure 4:**
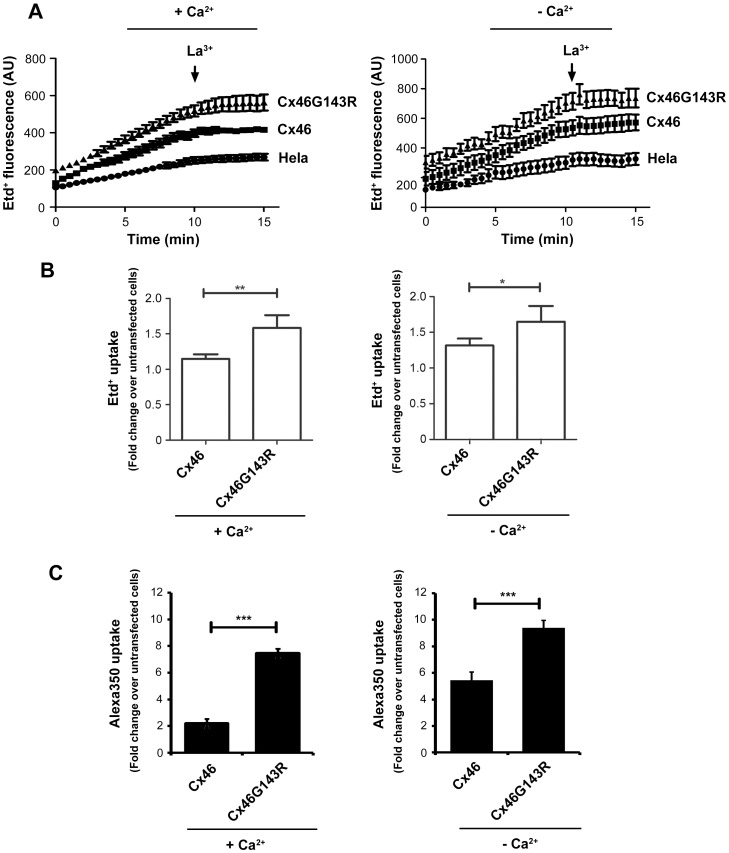
Cx46G143R increases hemichannel activity. *A)* Time lapse recording of Etd^+^ uptake in HeLa cells transfected with Cx46 or Cx46G143R. Cells were washed with recording media with (left panel) or without (right panel) 1.8 mM of CaCl_2_ 3 times and then incubated with 25 µM Etd^+^ for 15 min. During this time frame, fluorescence images were taken every 30 sec. LaCl_3_, a known connexin hemichannel blocker, was added after 10 min (arrow). *B)* Etd^+^ dye uptake or *C)* Alexa350 dye uptake was performed in HeLa cells stably transfected with Cx46 or Cx46G143R. Cells were washed with recording media with (left panel) or without (right panel) 1.8 mM of CaCl_2_ 3 times and then incubated with 50 µM mM Etd^+^ for 5 min or Alexa350 for 10 min. The intensity of Etd^+^ or Alexa350 fluorescence was measured and quantified. Untransfected HeLa cells were used as control. In absence or presence of extracellular Ca^2+^, Cx46G143R versus wild-type Cx46, ^*^, *P*<0.05; ^**^, *P*<0.01; ^***^, *P*<0.001. n = 5.

The hemichannels likely form in the presence of Ca^2+^, but are not open very much [14]; [15]. To determine if the G143R mutant affects Cx46 hemichannel activity, cellular dye uptake of ethidium bromide (Etd^+^) was assessed in absence and presence of Ca^2+^. We measured the uptake of 25 µM Etd^+^ by time lapse recording in untransfected, wild-type Cx46 or Cx46G143R transfected HeLa cells (Fig. 4A). In contrast to the intercellular spread of dye, the rate of Etd^+^ uptake by cells expressing the G143R mutant was higher than those expressing Cx46 or untransfected HeLa control cells in either high (left) or low Ca^2+^ (right) conditions. Comparison of the total uptake of Etd^+^ after 5 minutes revealed the same pattern (Fig. 4B). The increased hemichannel activity was further confirmed by using another fluorescence tracer dye, Alexa350 (Fig. 4C). The extent of the increase was even more significant than that determined by Etd^+^ uptake in both the absence and presence of 1.8 mM Ca^2+^. The difference between these two types of tracer dyes could be caused by the lower background (untransfected cells) uptake of Alexa350 dye. Interestingly, even under 1.8 mM Ca^2+^, the enhancement of hemichannel dye uptake was observed with Cx46G143R mutant.

**Figure 5:**
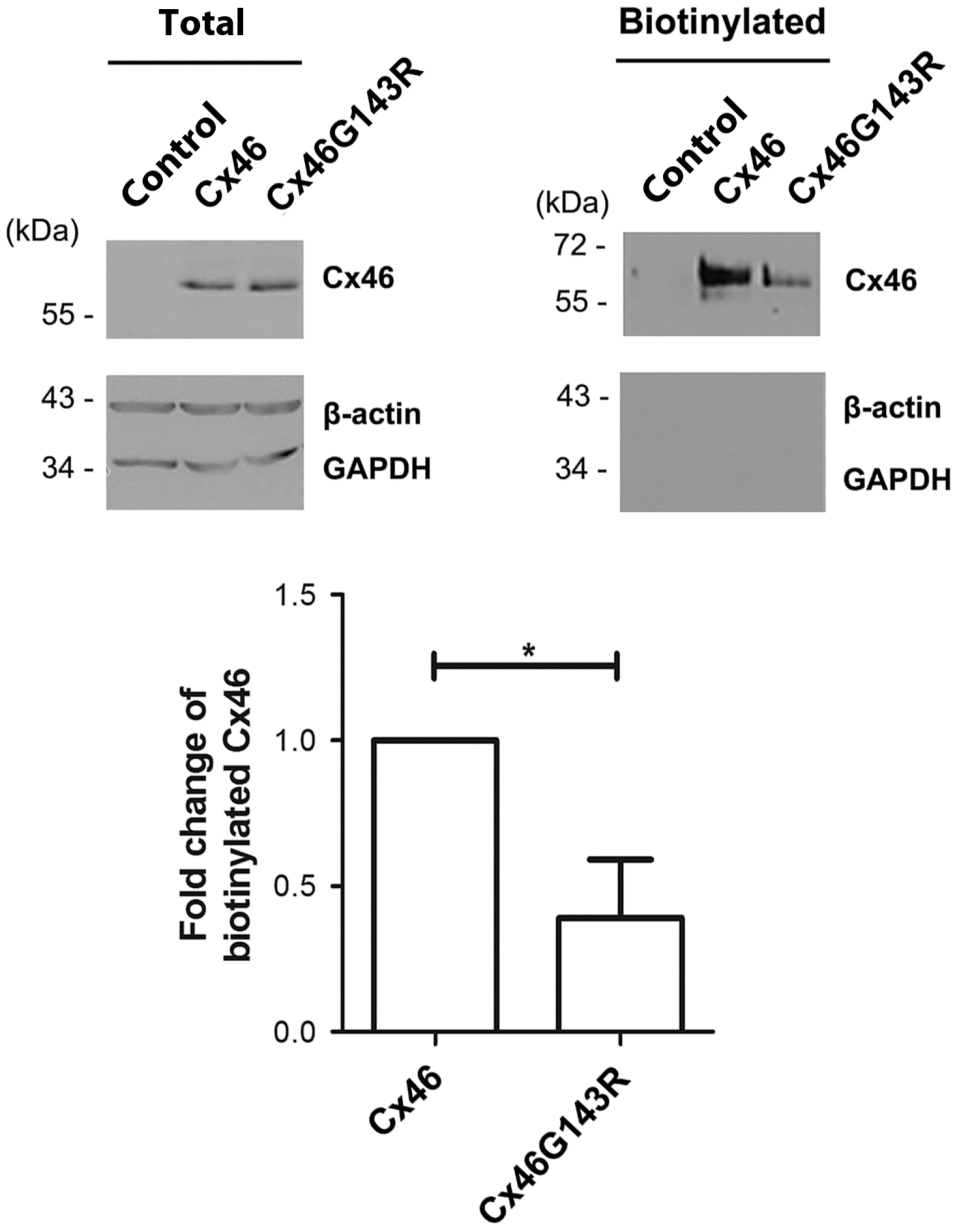
Cx46G143R decreases cell surface expression. HeLa cells stably transfected with Cx46, Cx46G143R, or untransfected controls were labeled with Sulfo-NHS-LC-Biotin. Equal amounts of total protein in cell lysates were mixed with neutravidin-conjugated avidin beads. The total lysate (left panel) and isolated biotinylated (right panel) proteins were analyzed by western blotting using anti-Cx46 antibody. β-actin and GAPDH were used as biotinylation controls. The bands of Cx46 were quantified using densitometry (NIH Image J software). The normalized ratios of biotinylated and total wild-type Cx46 or Cx46G143R were calculated (bottom panel). Cx46G143R versus Cx46, *, *P*<0.05. n = 4.

Immunofluorescence studies showed higher levels of mutant than wild-type Cx46 in plaque like structure between cells (confirmed by higher levels of detergent-resistant Cx46 in mutant cells). However, unlike gap junctions, hemichannels are located on the free cell surface, and not necessarily at sites of cell–cell apposition. They are also detergent-soluble. By the same token, they are readily accessible for surface biotinylation, which connexins in gap junctions are not due to the narrow extracellular space [13]. Thus, cell surface biotinylation should provide an estimate of connexin levels on the membrane available to form hemichannels. Surprisingly, less biotinylated Cx46 protein was detected in cells expressing the Cx46G143R mutant compared to wild-type Cx46 (Fig. 5, right panel). As negative controls, no biotinylated protein was detected for β-actin and GAPDH. By quantification of the ratio of biotinylated to total Cx46 or Cx46G143R, cell surface expressed wild-type Cx46 was two-fold higher than Cx46G143R (Fig. 5, bottom panel). As was the case with gap junctions, function seemed to be inversely correlated with protein levels, suggestive of effects on open probability of the channels rather than a more generic effect on expression levels or trafficking.

### G143R mutation increased the interaction between the intercellular loop domain and Cx46

**Figure 6:**
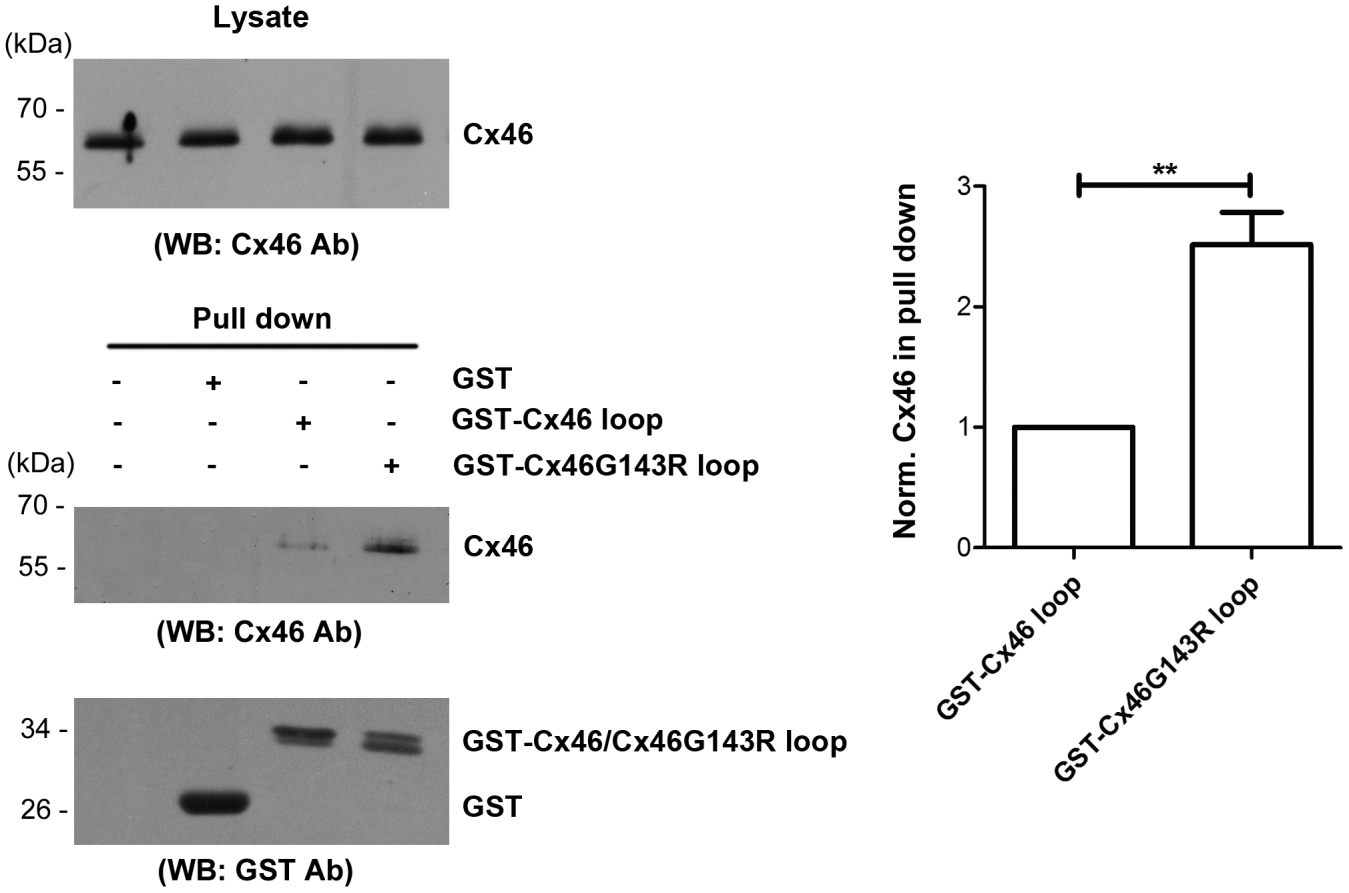
The G143R mutation increases the interaction between intracellular loop and Cx46. Crude membrane extract of cells expressing Cx46 were prepared and equal amount of Cx46 protein, as determined by immunoblotting with anti-Cx46 antibody (upper left panel), were incubated with glutathione beads conjugated with GST, GST-Cx46 loop or GST-Cx46G143R loop fusion proteins or beads alone. The eluted fractions of Cx46 from beads were immunoblotted with anti-Cx46 antibody, then stripped and reblotted with anti-GST antibody (lower left panels). The band intensity was quantified and the ratio of eluted (bound) Cx46 from wild-type and mutant Cx46 loop domains was calculated (right panel). Cx46G143R verses wild-type Cx46, ^**^, *P*<0.01. n = 3.

Since the cytoplasmic loop domain of Cx43 has been implicated in interactions with the C-terminal domain of this connexin, associated with gating [16]; [17], we conducted a pull down experiment, to test if these possible alterations of the intracellular loop domain of Cx46 could affect its interaction with its COOH-terminal domain. While both GST-Cx46 loop and GST-Cx46G143R loop domains, but not GST or glutathione-agarose beads, were able to pull down Cx46 from lysates of transfected HeLa cells (Fig. 6), the mutant was over 2 fold more efficient (Fig. 6, right panel).

### G143R mutation reduced cell viability and increased cell apoptosis

**Figure 7:**
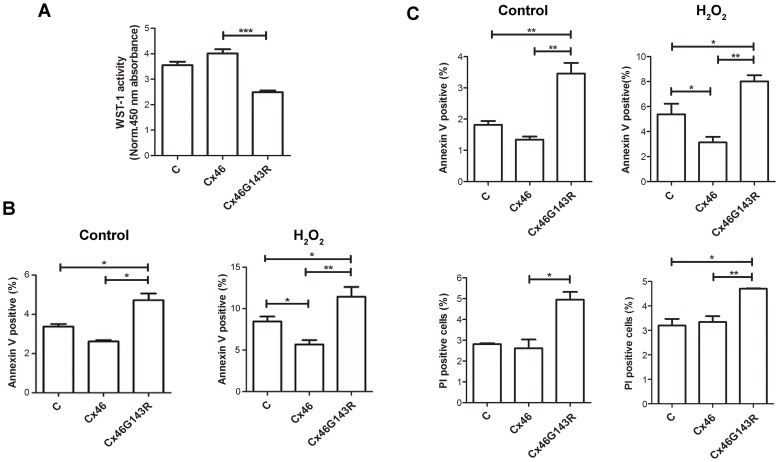
Cx46G143R decreases cell viability and promotes cell death. *A)* HeLa cells transfected with wild-type Cx46, Cx46G143R or untransfected control were incubated with WST-1 reagent and the absorbance at 450 nm was measured, which indicates the number of viable cells. The absorbance of the solution only containing culture medium and WST-1 reagent was measured as the background control. The absorbance of Cx46G143R versus that of Cx46, ^***^, *P*<0.001. n = 5. *B)* HeLa cells stably transfected with wild-type Cx46, Cx46G143R or non-transfected cells were treated with (right panel) or without (left panel) 0.5 mM H_2_O_2_. Twelve hr after treatment, cells at 90% confluency were labeled with annexin V and analyzed by flow cytometry to measure the percentage of annexin V positive cells. Cx46G143R versus Cx46, ^*^, *P*<0.05, ^**^, *P*<0.01. n = 5. *C)* Sparse cultured cells with minimal cell contacts were treated with (right panel), or without (left panel), 0.5 mM H_2_O_2_. Flow cytometry was used to measure the percentage of annexin V (upper panels) and PI (lower panels) positive cells. Cx46G143R versus Cx46, ^*^, *P*<0.05, ^**^, *P*<0.01. n = 3.

Given the effects of the G143R mutation on hemichannel opening, a WST-1 assay was used to determine if expression of Cx46G143R had any impact on cell viability. The absorbance at 12 hr after seeding was assessed as baseline to eliminate any effects on cell attachment. Expression of Cx46G143R significantly reduced the number of viable, attached cells as compared with the expression of wild-type Cx46 (Fig. 7A).

To further assess the effects of the G143R mutation on cells subjected to additional stress, cells were treated with and without 0.5 mM of H_2_O_2_. Annexin V labeling was used for detection of apoptotic cells and PI for necrotic cells. Flow cytometry showed that the number of annexin V-positive cells in Cx46G143R was increased compared to wild-type Cx46 in both H_2_O_2_-treated or non-treated control samples (Fig. 7B). However, there was no significant difference in PI-positive cells between wild-type Cx46 and Cx46G143R. This result suggests that expression of Cx46 increased cell viability and decreased cells under apoptosis under non- or oxidative stress conditions, while G143R mutation compromised the cell protective role of Cx46.

To distinguish the roles of gap junctions versus hemichannels, we examined the degree of apoptosis and necrosis in cells cultured at low density with minimal cell-cell contacts. The result showed that Cx46G143R expressed in sparsely cultured cells similarly increased cell apoptosis, as it did in confluent cells in the absence and presence of 0.5 mM H_2_O_2_ (Fig. 7C, upper panels). Unlike its effect on confluently cultured cells, this mutation also increased the overall number of dead cells as assessed by PI staining in sparely cultured cells (Fig. 7C, lower panels). These results show that Cx46G143R expression increased the levels of overall cell death even in sparse cultures where no significant gap junction coupling can occur.

## Discussion

In this study, we focused on a human mutation of a highly conserved residue in the Cx46 protein, the gene (*GJA*3) linked to human congenital Coppock cataracts in a four-generation Chinese family. This mutation is located in the intracellular loop domain, an uncommon site since most of human connexin mutations associated with disease occur in the extracellular loop and transmembrane domains. This mutation greatly reduced the ability of Cx46 to form functional gap junctions and had dominant negative characteristics in that this mutant inhibited the function of gap junctions formed by wild-type Cx46 when co-expressed. Contrary to its effects on gap junction function, the mutant seemed to enhance the formation of the gap junctional plaques. Therefore, the altered gap junction channels are not the consequences of assembly of junctional plaques. In contrast to its effects on gap junctions, the G143R mutation caused an increase in functional hemichannels, despite a drop in levels of freely, accessible (i.e. non-gap junctional) Cx46 on cell surface.

Most of these mutations occur in extracellular loop and the first/second transmembrane domains which have been associated with gap junction formation and pore structure/gating, respectively. Recently, another mutation in the cytoplasmic loop domain, V139M was identified in a 76-year-old male Chinese patient, although this patient had no family history of congenital cataracts, making it difficult to assess to what extent it contributed to the disease [18]. Thus this report is the first to associate a molecular mechanism of Cx46 mutation in the development of cataracts with the cytoplasmic loop domain.

We showed that Cx46G143R mutation had only about 50% dye coupling as compared to wild-type Cx46. The reduced gap junctional coupling was also reported in Cx43 mutation in the cytoplasmic loop. G138R and G143S mutants (non-homologous sites to G143 in Cx46) show minimal transfer of injected dye [19]. Partially in agreement with our result, a study reports that I130T mutation at intracellular loop domain of Cx43 reduces gap junctional dye transfer; however, this reduction is associated with the decreased formation of junctional plaques [20]. Likewise, another study demonstrates that a deletion of seven amino acids (Δ130-136) at intercellular loop domain of Cx43 loses its ability in forming gap junction channels and also plays a dominant negative role when co-expressed with wild-type Cx43 [21]. This deletion mutant also has normal expression pattern on the plasma membrane.

Cx46G143R mutant inhibited function of wild-type Cx46 gap junctions in a dominant negative manner, which is consistent with autosomal dominant inheritance pattern of congenital cataracts linked to this mutation [10]. The G143R mutant is able to assemble on cell-cell interfaces, which excludes the possibility that the impaired gap junctional coupling is due to abnormal delivery of Cx46 to junctional plaques. Since this mutant forms even larger junctional plaques than wild-type Cx46, a possible explanation could be that connexons formed by the mutant somehow may have even stronger interaction with those from neighboring cells. However, due to alteration of protein structure, the orientation of docking between mutants and mutants/wild-type could be tiled or twisted, resulting in reduced gap junction coupling and dominant negative effect. The conserved mutation G138R on Cx43 showed similar effect to that in Cx46, in that this mutant forms large gap junction plaque, but cannot transfer dye, and also functions in a dominant negative manner [22]; [23]. Additionally, a conserved threonine mutation in the cytoplasmic loop domain of multiple connexins, located 5 residues away from the mutant studied here (i.e. Cx43T154A, Cx26T135A, and Cx50T157C) showed an absence of dye coupling, but the presence of large gap junctional plaques [24].

Usually, increased hemichannel activity is associated with increased cell surface expression of connexin. However, we showed here that enhanced activity of hemichannels in Cx46G143R mutant was inversely correlated with cell surface expression levels of Cx46 protein, at least that portion which is not tied up in gap junctions and inaccessible for biotinylation. A previous study showed that the mutants in the cytoplasmic loop domain of Cx43, G143R and G143S, also have increased hemichannel opening under Ca^2+^-free conditions [19]. However, G143 in Cx43 is not the corresponding residue of G143 in Cx46, as the latter is G149 in Cx43. Another study shows that both N63S and fs380 impair the formation of hemichannels and gap junction channels, but did not affect gap junctional conductance when co-expressed with wild-type Cx46 [25]. Also, the N63Q mutant of Cx46 has been found to form functional hemichannels [26]. It has been known that hemichannels could be regulated by extracellular Ca^2+^ and Ca^2+^ removal results in an increase in the current of rat Cx46 hemichannels [26]. Similarly, reduction of external Ca^2+^ increases the open probability of hemichannels [27]. High extracellular Ca^2+^ keeps hemichannels in the closed state [28]. Here, we found even with close to 2 mM Ca^2+^, the hemichannel activity can be detected in cells expressing Cx46G143R. This mutant increases hemichannel activity, which could be an underlying cause for reduced cell viability.

Consistent with our study, it has been shown that some human connexin mutations linked to disease still form active channels but have altered permeability [29]; [30]. In these studies, similar fluorescence tracer dyes are used as we reported here. Therefore, the decreased coupling and increased hemichannel activity may actually reflect altered permeability of functioning channels. We used several fluorescence tracer dyes to validate our observations. In addition to Etd^+^ and Alexa 350, we also used Lucifer yellow and biocytin; however, the latter two dyes were not permeable to human Cx46 forming channels (data not shown).

The single-nucleotide change from G to R led to the replacement of small neutral amino acid with a larger, positively charge residue. The glycine at position 143 is highly conserved across species and human connexins. The similar region in Cx43 (amino acid 119 to 144) has been shown to contribute to the direct interactions between intracellular loop and C-terminus, and this interaction is critical for gap junction and hemichannel function [17]. In addition, this region of the intracellular loop domain of Cx43 has been found to interact with C-terminus which also mediates the closure of gap junction channels [31]. It has been proposed that the cytoplasmic loop may serve as a receptor for the C-terminus that regulates the permeability of gap junctional channels through “ball-and-chain” mechanism [16]; [32]. However, this interaction appears to play an opposite role in hemichannel function. The binding of C-terminus to intracellular loop of Cx43 was considered an essential step to render Cx43 hemichannels in an active state [17]. Both Cx46 and Cx43 belong to gap junction α; a protein family with regard to their similar sequence and length of the cytoplasmic domains [33]. It is likely that a similar region on Cx46 plays a similar role for gap junction function as it does in Cx43. Indeed, our results support this assertion. We showed that the G143R mutation increased the interaction between the intracellular loop domain and Cx46, which is associated with the reduction of gap junction but increase of hemichannel function.

The reduction of cell viability in cell expressing Cx46 G143R could be potentially caused by the decreased gap junctions and/or increased hemichannel activity. However, since the decreased viability and increased apoptosis of the cells expressing the G143R mutant is more prominent than in non-connexin expressing control cells, the effect of the mutant is likely associated with a gain of function, implicating the abnormal increase of hemichannel activity associated with the mutant. In support of this, cells cultured at low density with minimal gap junctions also showed the similar degree of cell viability and cells under apoptosis as cells cultured at normal density. “Leaky” hemichannels have been reported to cause abnormal depolarization of membrane potential, disruption of intracellular microenvironment and ultimately cell death [34]. A G46V mutation in Cx50, identified in a child of Palestinian origin with cataract, forms normal gap junctions, but increases hemichannel activity [35]. Similarly, the increased hemichannel activity of this mutant is associated with decreased cell viability. Oxidative stress causes the damage in lens associated with increased cell apoptosis and necrosis, and contributes to the development of various types of cataracts [36]. We showed that expression of wild-type Cx46 protected cells against H_2_O_2_-induced cell death. However, this cell protective role of Cx46 is significantly compromised with G143R mutation. Since connexin channels play essential roles in maintaining lens cell homeostasis, metabolic coupling and preventing accumulation of reactive oxidants, abnormal activities of gap junctions and/or hemichannels would greatly compromise these important functions and consequently, cell death occurs associated with cataract formation.

## References

[1] Forrester J, Dick A, Mcmenamin P, Lee W (1996) The eye: basic sciences in practice, W.B. Saunders Company Ltd, London

[2] Mathias RT, White TW, Gong X (2010) Lens gap junctions in growth, differentiation, and homeostasis. Physiol.Rev. 90: 179–206. 10.1152/physrev.00034.2009 PMC4627646 20086076

[3] Goodenough DA (1992) The crystalline lens: a system networked by gap junctional intercellular communication. In Gilula, N. B., editor. Seminars in Cell Biology, Sauders Scientific Pub./Academic Press, London 10.1016/s1043-4682(10)80007-8 1320431

[4] Goodenough DA, Goliger JA, Paul DL (1996) Connexins, connexons, and intercellular communication. Annu.Rev.Biochem. 65: 475–502. 10.1146/annurev.bi.65.070196.002355 8811187

[5] Mese G, Richard G, White TW (2007) Gap junctions: Basic structure and function. J.Invest.Dermatol. 127: 2516–2524. 10.1038/sj.jid.5700770 17934503

[6] Mathias RT, Kistler J, Donaldson P (2007) The lens circulation. J.Membr.Biol. 216: 1–16. 10.1007/s00232-007-9019-y 17568975

[7] Gong X, Li E, Klier G, Huang Q, Wu Y, et al (1997) Disruption of a_3_ connexin gene leads to proteolysis and cataractogenesis in mice. Cell 91: 833–843. 9413992 10.1016/s0092-8674(00)80471-7

[8] Jiang JX (2010) Gap junctions or hemichannel-dependent and independent roles of connexins in cataractogenesis and lens development. Curr.Mol.Med. 10: 851–863. 10.2174/156652410793937750 PMC6263138 21091421

[9] Bennett TM, Shiels A (2011) A recurrent missense mutation in GJA3 associated with autosomal dominant cataract linked to chromosome 13q. Mol.Vis. 17: 2255–2262. PMC3164684 21897748

[10] Zhang L, Qu X, Su S, Guan L, Liu P (2012) A novel mutation in GJA3 associated with congenital Coppock-like cataract in a large Chinese family. Mol.Vis. 18: 2114–2118. PMC3413429 22876138

[11] Nettleship E, Ogilvie FM (1906) A peculiar form of hereditary congenital cataract. Trans.Ophthalmol.Soc.UK 26: 191–206.

[12] Liu J, Ek Vitorin JF, Weintraub ST, Gu S, Shi Q, et al (2011) Phosphorylation of connexin 50 by protein kinase A enhances gap junction and hemichannel function. J.Biol.Chem. 286: 16914–16928. 10.1074/jbc.M111.218735 PMC3089535 21454606

[13] Musil LS, Goodenough DA (1991) Biochemical analysis of connexin43 intracellular transport, phosphorylation, and assembly into gap junctional plaques. J.Cell Biol. 115: 1357–1374. 10.1083/jcb.115.5.1357 PMC2289231 1659577

[14] Paul DL, Ebihara L, Takemoto LJ, Swenson KI, Goodenough DA (1991) Connexin46, a novel lens gap junction protein, induces voltage- gated currents in nonjunctional plasma membrane of Xenopus oocytes. J.Cell Biol. 115: 1077–1089. 10.1083/jcb.115.4.1077 PMC2289939 1659572

[15] Ebihara L, Tong JJ, Vertel B, White TW, Chen TL (2011) Properties of connexin 46 hemichannels in dissociated lens fiber cells. Invest.Opthalmol.Vis.Sci. 52: 882–889. 10.1167/iovs.10-6200 PMC3053112 20861491

[16] Morley GE, Taffet SM, Delmar M (1996) Intramolecular interactions mediate pH regulation of connexin43 channels. Biophys.J. 70: 1294–1302. 10.1016/S0006-3495(96)79686-8 PMC1225055 8785285

[17] Ponsaerts R, de Vuyst E, Retamal M, D′hondt C, Vermeire D, et al (2010) Intracellular loop/tail interaction are essential for connexin 43-hemichannel activity. FASEB J. 24: 4378–4395. 10.1096/fj.09-153007 20634352

[18] Zhou Z, Wang B, Hu S, Zhang C, Ma X, et al (2011) Genetic variations in GJA3, GJA8, LIM2, and age-related cataract in the Chinese pupulation: a mutation screening study. Mol.Vis. 17: 621–626. PMC3049737 21386927

[19] Dobrowolski R, Sommershof A, Willecke K (2007) Some oculodentodigital dysplasia-associated Cx43 mutations cause increased hemichannel activity in addition to deficent gap junction channels. J.Membrane Biol. 219: 9–17. 10.1007/s00232-007-9055-7 17687502

[20] Lai A, Le DN, Paznekas WA, Gifford WD, Jabs EW, et al (2006) Oculodentodigital dysplasia connexin43 mutations results in non-functional connexin hemichannels and gap junctions in C6 glioma cells. J.Cell Sci. 119: 532–541. 10.1242/jcs.02770 16418219

[21] Krutovskikh V, Yamasaki H, Tsuda H, Asamoto M (1998) Inhibition of intrinsic gap-junction intercellular communication and enhancement of tumorigenicity of the rat bladder carcinoma cell line BC31 by a dominant negative connexin 43 mutant. Mol.Carcinog. 23: 254–261. 9869455

[22] Roscoe W, Veitch GI, Gong XQ, Pellegrino E, Bai D, et al (2005) Oculodentodigital dysplasia-causing connexin43 mutants are non-functional and exhibit dominant effects on wild-type connexin43. J.Biol.Chem. 280: 11458–11466. 10.1074/jbc.M409564200 15644317

[23] Gong X, Cheng C, Xia C (2007) Connexins in lens development and cataractogenesis. J.Memb.Biol. 218: 9–12. 10.1007/s00232-007-9033-0 17578632

[24] Beahm DL, Oshima A, Gaietta GM, Hand GM, Smock AE, et al (2006) Mutation of a conserved threonine in the third transmembrane helix of alpha- and beta-connexins creates a dominant-negative closed gap junction channel. J.Biol.Chem. 281: 7994–8009. 10.1074/jbc.M506533200 16407179

[25] Pal JD, Liu X, Mackay D, Shiels A, Berthoud VM, et al (2000) Connexin46 mutations linked to congenital cataract show loss of gap junction channel function. Am.J.Physiol.(Cell Physiol.) 279: C596–C602. 10942709 10.1152/ajpcell.2000.279.3.C596

[26] Ebihara L, Liu X, Pal JD (2003) Effect of external magnesium and calcium on human connexin 46 hemichannels. Biophys.J. 84: 277–286. 10.1016/S0006-3495(03)74848-6 PMC1302609 12524281

[27] Pfahnl A, Dahl G (1999) Gating of cx46 gap junction hemichannels by calcium and voltage. Eur.J.Physiol. 437: 345–353. 10.1007/s004240050788 9914390

[28] Müller DJ, Hand GM, Engel A, Sosinsky GE (2002) Conformational changes in surface structures of isolated connexin 26 gap junctions. The EMBO J. 21: 3598–3607. 10.1093/emboj/cdf365 PMC126111 12110573

[29] Xu J, Nicholson BJ (2013) The role of connexins in ear and skin physiology - functional insights from disease-associated mutations. Biochim.Biophys.Acta 1828: 167–178. 22796187 10.1016/j.bbamem.2012.06.024 PMC3521577

[30] Pfenniger A, Wohlwend A, Kwak BR (2011) Mutations in connexin genes and disease. Eur.J Clin.Invest 41: 103–116. 20840374 10.1111/j.1365-2362.2010.02378.x

[31] Seki A, Duffy HS, Coombs W, Spray DC, Taffet SM, et al (2004) Modification in the biophysical properties of connexin43 channels by a peptide of the cytoplasmic loop region. Circ.Res. 95: e22–28. 10.1161/01.RES.0000140737.62245.c5 15284189

[32] Duffy HS, Sorgen PL, Girvin ME, O′Donnell P, Coombs W, et al (2002) pH-depdendent intramolecular binding and structure involving Cx43 cytoplasmic domains. J.Biol.Chem. 277: 36706–36714. 10.1074/jbc.M207016200 12151412

[33] Söhl G, Willecke K (2003) An update on connexin genes and their nomenclature in mouse and man. Cell Commun.Adhes. 10: 173–180. 10.1080/cac.10.4-6.173.180 14681012

[34] Contreras JE, Sanchez HA, Veliz LP, Bukauskas FF, Bennett MV, et al (2004) Role of connexin-based gap junction channels and hemichannels in ischemia-induced cell death in nervous tissue. Brain Res.Rev. 2004: 290–303. 10.1016/j.brainresrev.2004.08.002 PMC3651737 15572178

[35] Minogue PJ, Tong JJ, Arora A, Russell-Eggitt I, Hunt DM, et al (2009) A mutant connexin50 with enhanced hemichannel function leads to cell death. Invest.Ophthal.Vis.Sci. 50: 5837–5845. 10.1167/iovs.09-3759 PMC2788668 19684000

[36] Berthoud VM, Beyer EC (2009) Oxidative stress, lens gap junctions, and cataracts. Antioxid.Redox Signal. 11: 339–353. 10.1089/ars.2008.2119 PMC2763361 18831679

